# Osteoblasts and osteoclasts: an important switch of tumour cell dormancy during bone metastasis

**DOI:** 10.1186/s13046-022-02520-0

**Published:** 2022-10-28

**Authors:** Rongchen Dai, Mengfan Liu, Xincheng Xiang, Zhichao Xi, Hongxi Xu

**Affiliations:** 1 School of Pharmacy, Shanghai University of Traditional Chinese Medicine, Shanghai, 201203 China; 2 Engineering Research Center of Shanghai Colleges for TCM New Drug Discovery, Shanghai, 201203 China; 3 Rausser College of Natural Resources, University of California Berkeley, Berkeley, CA 94720 USA; 4 Shuguang Hospital, Shanghai University of Traditional Chinese Medicine, Shanghai, 201203 China

**Keywords:** Tumour cell dormancy, Osteoclasts, Osteoblasts, Bone marrow niche, Bone metastasis

## Abstract

Bone metastasis occurs when tumour cells dissociate from primary tumours, enter the circulation (circulating tumour cells, CTCs), and colonize sites in bone (disseminated tumour cells, DTCs). The bone marrow seems to be a particularly dormancy-inducing environment for DTCs, yet the mechanisms of dormancy initiation, reactivation, and interaction within the bone marrow have to be elucidated. Intriguingly, some evidence has suggested that dormancy is a reversible state that is switched ‘on’ or ‘off’ depending on the presence of various bone marrow resident cells, particularly osteoclasts and osteoblasts. It has become clear that these two cells contribute to regulating dormant tumour cells in bone both directly (interaction) and indirectly (secreted factors). The involved mechanisms include TGFβ signalling, the Wnt signalling axis, the Notch2 pathway, etc. There is no detailed review that specifically focuses on ascertaining the dynamic interactions between tumour cell dormancy and bone remodelling. In addition, we highlighted the roles of inflammatory cytokines during this ‘cell-to-cell’ communication. We also discussed the potential clinical relevance of remodelling the bone marrow niche in controlling dormant tumour cells. Understanding the unique role of osteoclasts and osteoblasts in regulating tumour dormancy in bone marrow will provide new insight into preventing and treating tumour bone metastasis.

## Background

Tumour metastasis remains an extraordinarily complicated and poorly understood process. During tumour metastasis, cancer cells first undergo epithelial-to-mesenchymal transition (EMT) to dissociate from the primary tumour and enter the microvasculature of the blood and lymphatic systems [1]. Once entering the circulation, circulating tumour cells (CTCs) may extravasate from the blood vessels, disseminate to distant organs and settle in the secondary microenvironment, where they are termed disseminated tumour cells (DTCs) [2, 3]. It is thought that the majority of DTCs from different primary tumours are inclined to be delivered to the bone, because the bone marrow is an especially congenial soil for tumour cell metastasis due to its rich sources of growth factors, neovascularization factors, cytokines, and chemokines [4]. Previous clinical observation revealed that bone metastasis only forms in sites that host haematopoietically active red marrow [5]. One of the possible reasons is DTCs are collected by the spongy tissue of red marrow that normally contains haematopoietic stem cells (HSCs) during bone metastasis [6]. However, there are considerable barriers to cell colonization and growth in the bone microenvironment, and the physical properties of bones make them a harsh and unwelcoming site for colonizing DTCs [7].

To survive and grow, cancer cells must navigate themselves to adapt to these features in the bone microenvironment, thus it is sensible for DTCs to enter the dormant state. Tumour dormancy is generally divided into two categories, tumour cell dormancy and tumour mass dormancy. Tumour cell dormancy (also termed as quiescent cancer cells) refers to cancer cells, including CTCs and DTCs, that are transiently suspended in G_0_ phase, and able to re-enter the cell cycle and re-proliferate [8]. While tumour mass dormancy is characterized by insufficient angiogenesis and immune surveillance [9]. Dormant tumour cells are therapeutically challenging owing to their resistance to most radio-chemotherapies that target proliferative cancer cells [10]. Moreover, tumour cell dormancy could resist CD8+ T cell attack by orchestrating a local hypoxic immune-suppressive milieu [11]. Indeed, most of the colonizing DTCs were found in a dormant state instead of proliferating when they anchored the bone surface [12–14]. Because endosteal bone surface is a predominant (approximately 80%) dormancy-inducing microenvironment, which consists of quiescent bone lining cells, adipocytes, osteomas, immune cells and neurocytes [7, 15]. After years or even decades, these dormant tumour cells may reactivate, reproliferate to subclinical growth and eventually become detectable as a late metastatic relapse [16]. Notably, when solid tumours metastasize to the bone, they are often considered incurable. Therefore, there is an urgent need to expand our understanding of the cellular and molecular interactions between dormant tumour cells and the bone microenvironment and to develop therapies to prevent tumour bone metastasis.

Accumulating evidence has revealed the close involvement of bone remodelling during the progression of bone metastasis. Bone remodelling involves a complex set of interactions that result in an overall maintenance of bone mass or an anabolic or catabolic bone state [17]. This dynamic equilibrium is well controlled by a diverse population of bone marrow resident cells, such as osteoblasts, osteoclasts, bone lining cells, and osteocytes. Particularly, attention should be devoted to osteoblast-mediated bone formation and osteoclast-mediated bone resorption, two primary processes under dynamic balance that contribute to physiological bone remodelling [18]. Once the delicate balance is disturbed, diseases such as osteoporosis (excess bone loss) and osteopetrosis (excess bone formation), will occur [19]. More importantly, these bone diseases will further create a more favourable bone metastasis microenvironment for a diverse of primary tumour types.

Intriguingly, evidence has illustrated the unusual role of osteoblasts and osteoclasts in controlling the switch between dormancy versus proliferation in DTCs during bone metastasis [20]. Take myeloma as an example, dormancy would be initiated and maintained (dormancy switched on) by osteoblasts, but would be reactivated to proliferate (dormancy switched off) by osteoclasts [21]. A similar phenomenon also occurs in breast tumour dormancy [22] and prostate tumour dormancy [20], yet the underlying mechanisms are quite different and remain elusive. It is likely that osteoclasts and osteoblasts can either directly interact with dormant tumour cells or secrete various factors to control the dormant state of the tumour cells near the endosteal bone surface [23]. Conversely, dormant tumour cells could recruit osteoclast progenitors and enhance local osteoclast activity to reactivate them from dormancy [24], indicating that the ‘on-and-off dormancy switch’ of osteoblasts/osteoclasts could also be influenced by dormant tumour cells.

Several critical mechanisms involved in osteoclasts/osteoblasts and tumour cell dormancy must be elucidated. a. How do tumour cells initiate and maintain a dormant state via osteoclasts/osteoblasts in the bone? b. What factors released during bone remodelling trigger the reactivation of dormant tumour cells? c. Do dormant tumour cells influence the effects of the bone marrow niche on regulating tumour dormancy? In this review, we clarified and summarized the unique role of osteoclasts and osteoblasts in regulating tumour dormancy in bone marrow, highlighted the link of dormant tumour cells in remodelling the bone marrow niche, and discussed promising therapeutic approaches.

## Main text

### Bone formation initiates and maintains tumour cell dormancy

**Fig. 1:**
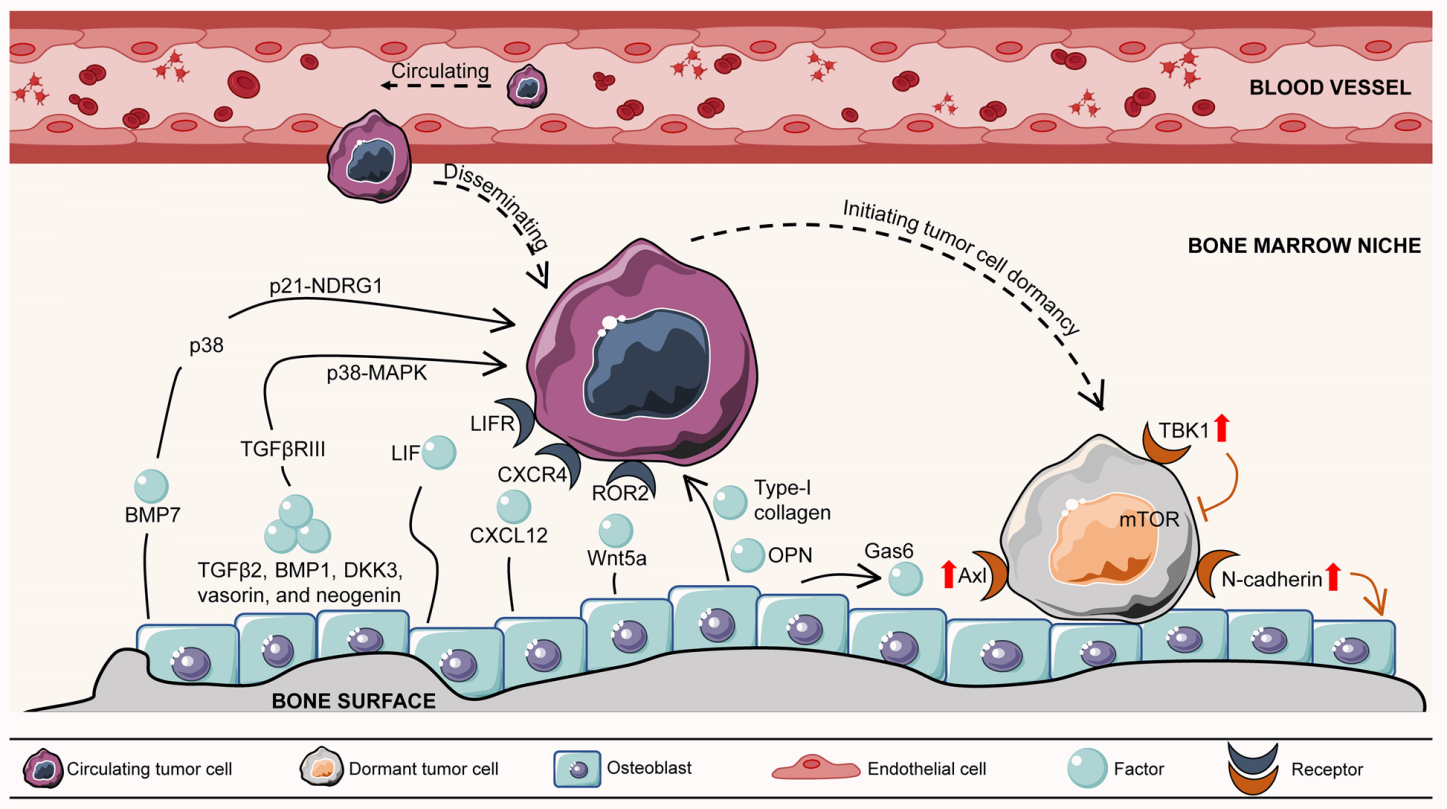
Osteoblast-mediated bone formation initiates and maintains tumour cell dormancy

Recent findings revealed the role of osteoblasts in promoting tumour cell dormancy through direct interactions with cancer cells (Table 1) (Fig. 1). By using a specialized 3D model of a bone mimic that permits the growth of a multiple layer of mineralized osteoblast tissue from pre-osteoblasts, human MCF-7 breast cancer cells were shown to enter a dormant state when co-cultured with normal human osteoblasts [25]. Moreover, human-derived osteoblast-like cells appeared to induce a dormant phenotype by downregulating migration- and proliferation-related proteins such as BMI1 and ID1 in primary breast cancer 3384T cells [22]. Capulli et al. revealed that spindle-shaped N-cadherin+/CD45- osteoblasts (SNOs) induced breast tumour cell dormancy through a Notch2-dependent mechanism in highly metastatic human MDA-MB-231 and mouse 4T1 cell lines. Instead, the inhibitory effect on cell division was not obvious in the cocultures of poorly-aggressive human breast cancer MCF-7 cells with SNOs. This result suggested that SNO-induced quiescence is a selective and specific occurrence that is likely associated with poor-prognosis breast tumours [26].

**Table 1:** The role of osteoblasts and osteoblastic niche in inducing tumour cell dormancy

Cancer types	Osteoblast-secreted factors	Mechanisms	Reference
Human breast cancer cells	Direct interaction	N/A	[25]
Primary breast cancer cells		↑ KLF7, THY1, PECA1, and PLAUR; ↓ BMI1 and ID1	[22]
Breast cancer cells		↑ Notch2 signalling	[26]
Myeloma cells	Type-I collagen	N/A	[21]
Primary leukaemia cells	OPN	↑ cell cycle exit	[23]
Prostate cancer cells	BMP7	↑ NDRG1 mRNA expression via activating p38 and p21	[27]
Prostate cancer cells	Wnt5a	↑ SIAH2/ROR2 signalling axis; ↓ Wnt/β-catenin signalling	[28]
Prostate cancer cells	TGFβ2	↑ TGFβRIII signalling; ↑ p38MAPK phosphorylation and nuclear translocation; ↑ pS249/pT252-RB in the nucleus; ↑ p27 and G_1_-cell cycle arrest	[20]
Breast cancer cells	LIF	Binding to LIFR and ↑ gene expressions of TSP1, TPM1, TGFβ2, P4HA1, miR-190 and SELENBP1	[29]
Cancer cells	CXCL12	Binding to CXCR4 in tumour cells and ↑ drug resistance	[30, 31]
Prostate cancer cells	Gas6	↑ Axl, Sky and Mer	[32]

*Note*: ‘↑’ represents increased, upregulated, induced, enhanced and activated; While ‘↓’ represents decreased, downregulated, inhibited; *N/A* represents not applicable

Apart from direct interaction between osteoblasts and cancer cells, it has also been observed that factors such as type-I collagen, osteopontin, and Wnt5a produced by osteoblasts or the osteoblastic niche could support tumour cell dormancy. Lawson and colleagues tracked individual myeloma cells by intravital imaging in a green fluorescent protein (GFP) transgenic mouse model. They found that dormant myeloma cells localized directly adjacent to endosteal bone surfaces occupied by type-I collagen-expressing osteoblast cells, while proliferating myeloma cells were preferentially found at locations distant from the bone surface [21]. Another important protein expressed by endosteal osteoblasts is osteopontin (OPN), a soluble cytokine or chemokine as well as an adhesive component of the extracellular matrix. After engrafting dormant primary leukaemia cells via the tail vein into NOD-SCID IL2Rγ(null) (NSG) mice, the majority of dormant cells were specifically present in high OPN expression regions of the calvarial bone marrow. Confocal analysis showed that a large number of dormant leukaemia cells directly colocalized with OPN. This interaction induced cell cycle exit in leukaemic blasts, protecting them from cytotoxic chemotherapy, while inhibiting the OPN signalling axis led dormant lymphoblastic leukaemia cells to proliferate, thereby sensitizing them to chemotherapy [23]. In addition, bone morphogenetic protein 7 (BMP7) secreted by osteoblasts induced the dormancy of PC-3 prostate cancer cells by upregulating the mRNA expression of N-myc downstream-regulated gene 1 (NDRG1), a metastasis suppressor gene, via activating p38 and p21. An *in vivo* study showed that withdrawal of BMP7 significantly abrogated the suppressive effect of osteoblasts and induced metastatic growth of stem-like prostate cancer cells in the bone [27]. Wnt signalling within the bone microenvironment plays a crucial role in the equilibrium of cell dormancy and reactivation [33]. Ren et al. revealed that Wnt5a produced from the osteoblastic niche induced bone metastatic prostate tumour cell dormancy by activating the receptor tyrosine kinase-like orphan receptor 2 (ROR2)/SIAH2 signalling axis, resulting in repression of the Wnt/β-catenin pathway. Silencing Wnt5a restored the growth ability of prostate tumour cells, indicating a potential therapeutic role of Wnt5a in preventing bone metastatic recurrence by inducing cancer cell dormancy [28].

Interestingly, the bone-derived transforming growth factor (TGF) β1 and TGFβ2 have been demonstrated to exert opposite functions on the behaviour of tumour cell dormancy in the bone marrow. TGFβ1 facilitates rapid tumour proliferation [34], while TGFβ2 promotes tumour cell dormancy. The expression of TGFβ2 secreted by osteoblasts was markedly upregulated during osteoblast differentiation and induced prostate tumour dormancy *in vitro* and *in vivo*, indicating that osteoblast differentiation may affect tumour dormancy [20]. Mechanistically, TGFβ2 secreted by differentiated osteoblasts activated TGFβRIII to stimulate p38 mitogen-activated protein kinases (MAPK) phosphorylation and nuclear translocation. Nuclear p-p38 then phosphorylates the N-terminus of retinoblastoma (RB) at Ser249/Thr252, leading to increased p27 and G_1_-cell cycle arrest [20]. Similar studies confirmed that activating the p38MAPK pathway by osteoblast-secreted factors, such as BMP1, dickkopf-related protein 3 (DKK3), vasorin, and neogenin [35], might drive tumour cells to a dormant state [36].

Currently, emerging evidence has proven that osteoblast-mediated bone formation and the related osteoblastic niche play a supportive role in dormancy induction and maintenance. This is contrary to previous beliefs that osteoblasts could encourage tumour growth by providing essential growth factors [37, 38]. To date, how osteoblasts determine whether to promote the growth of tumour cells or initiate and maintain them in dormancy remains largely unknown. Some views believe that the state of osteoblasts (active or quiescent) might be one of the reasons. In addition, the functions of different types of osteoblasts vary, which should also be taken into consideration in further investigations.

Osteoblasts can either interact directly with dormant tumour cells or indirectly through secretion of various factors, wherein both ways control the rate of cell proliferation and induce dormancy of the tumour cells near the bone surface. Osteoblast-secreted factors include BMP7, TGFβ2, BMP1, DKK3, vasorin, neogenin, LIF, CXCL12, Wnt5a, Type-I collagen, OPN, and Gas6. In turn, dormant tumour cells preferentially adhere to osteoblasts, thus facilitating bone formation to induce and maintain themselves in a dormant state. In this case, dormant tumour cells upregulate their expression of several signalling receptors, such as Axl, TBK1, and N-cadherin, thereby allowing adhesive attraction of dormant tumour cells to osteoblasts. BMP7, bone morphogenetic protein 7; BMP1, bone morphogenetic protein 1; TGF, transforming growth factor; DKK3, dickkopf-related protein 3; ROR2, receptor tyrosine kinase-like orphan receptor 2; NDRG1, N-myc downstream-regulated gene 1; MAPK, mitogen-activated protein kinases; LIF, leukaemia inhibitory factor; LIFR, LIF receptor; CXCL12, C-X-C motif chemokine ligand 12; CXCR4: C-X-C motif chemokine receptor 4; OPN, osteopontin; Gas6, growth-arrest specific 6; TBK1, TANK binding kinase 1; mTOR, mammalian/mechanistic target of rapamycin. Graphics were partly generated using Servier Medical Art, provided by Servier, licensed under a Creative Commons Attribution 3.0 unported license (https://smart.servier.com/).

### Bone resorption reactivates dormant tumour cells

**Fig. 2:**
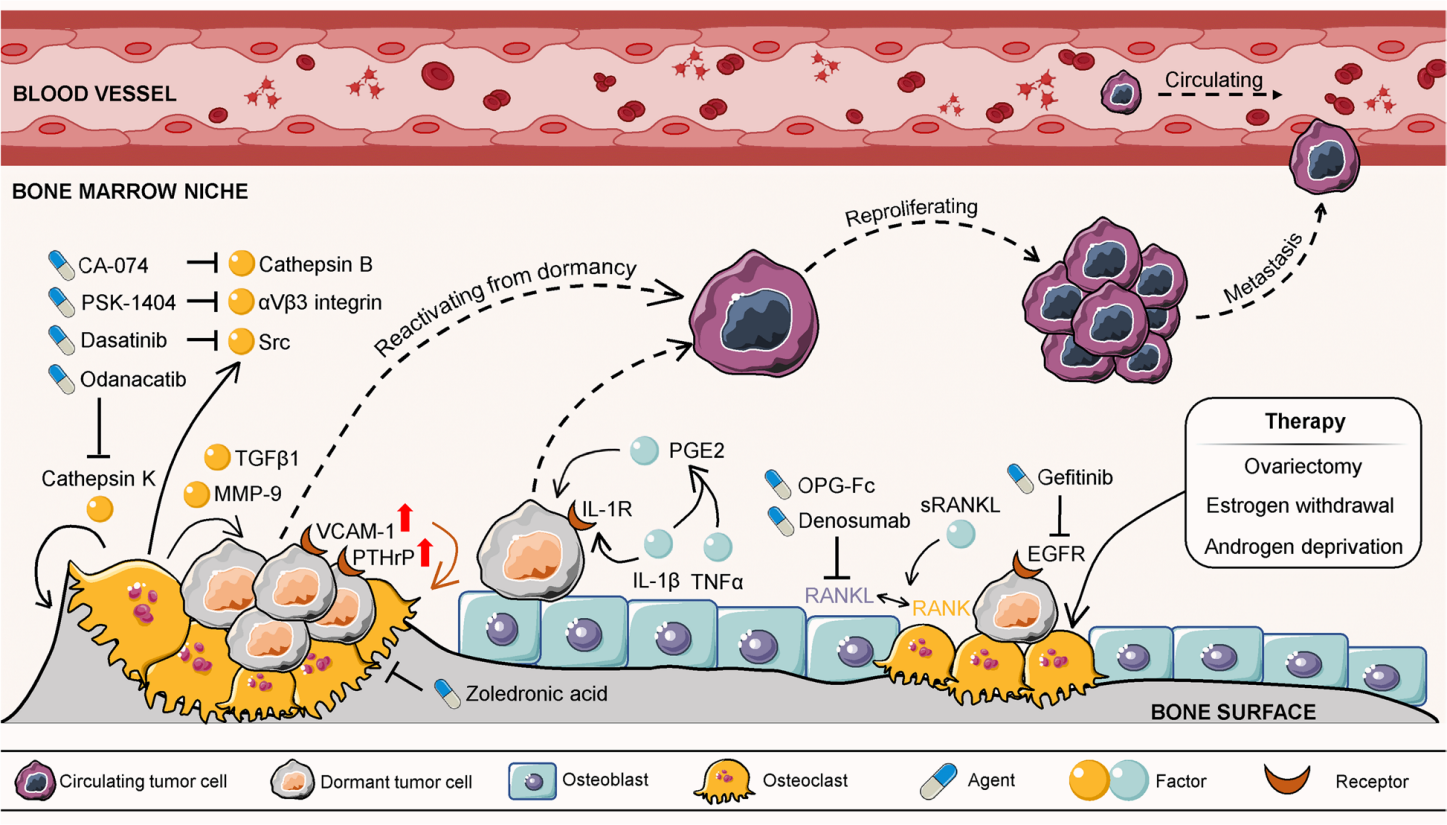
Osteoclast-mediated bone resorption reactivates dormant tumour cells

Tumour recurrence that occurs years after seemingly successful treatment of primary tumours is one of the major causes of mortality in cancer patients. The reactivation of dormant tumour cells is mainly responsible for this phenomenon. Therefore, blocking dormant tumour cells from reproliferation would be a promising strategy for preventing tumour relapse. Evidence has shown that bone metastasis tumour cells exit dormancy and can be influenced by factors produced during bone resorption (Table 2) (Fig. 2). In experimental models, increasing bone resorption through parathyroid hormone (PTH) stimulation or calcium restriction aggravated tumour development in bone [39, 40]. Cackowski et al. demonstrated that bone resorption stimulated bone angiogenesis in foetal mouse metatarsal explants by producing matrix metalloproteinase-9 (MMP-9) [41], a factor that could awaken dormant cancer cells through extracellular matrix (ECM) remodelling *in vivo* [42]. Moreover, TGFβ1, an important growth factor, is abundantly released from resorbed bone and activated by osteoclasts. In contrast to the role of TGFβ2 in osteoblast-induced dormancy, activated TGFβ1 could induce a mesenchymal phenotype and reawaken dormant breast tumour cells to rapid growth in the bone marrow [43]. Lawson et al. demonstrated that inducing bone resorption by a soluble form of the ligand for the receptor activator of NFκB (sRANKL) reactivated dormant myeloma cells from a proliferative-suppressed condition caused by osteoblasts or bone lining cells [21]. These results suggest that therapies that inhibit bone resorption might be beneficial for preventing tumour relapse by blocking dormant tumour cell reactivation.

**Table 2:** The role of osteoclasts and bone resorption in reactivating dormant tumour cells

Dormant cancer types	Factors that induced bone resorption	Mechanisms	Reference
Prostate and breast cancer cells	PTH stimulation / calcium restriction	↑ tumour development in bone	[39, 40]
Breast cancer cells	Osteoclasts secreted MMP-9	↑ bone angiogenesis and ECM remodelling	[41, 42]
Breast cancer cells	Osteoclasts secreted TGFβ1	↑ a mesenchymal phenotype and motility	[43]
Myeloma cells	Osteoblast expressed sRANKL	Remodelling the endosteal niche	[21]
Breast cancer cells	OVX (surgery)	↓ expression of the osteoblast formation inhibitors Dkk1, 2 and 3; ↑ PTH expression	[44]
ER-positive breast cancer cells	Oestrogen depletion (surgery)	↑ ANGPT2 signalling	[45]
Prostate cancer cells	Androgen deprivation (surgery)	↑ serum levels of the osteoclast marker TRAP and P1NP	[46]

*Note*: ‘↑’ represents increased, upregulated, induced, enhanced and activated; While ‘↓’ represents decreased, downregulated, inhibited

Bone resorption is also mediated by hormones, especially oestrogen and androgen, which might subsequently impact dormant tumour reactivation in bone (Table 2). Bone loss in women occurs rapidly in the years following menopause or experiencing ovariectomy (OVX) when natural levels of oestrogen are greatly reduced [47]. Ottwell and colleagues showed that increased bone resorption stimulated dormant breast tumours to proliferate. They found that the ratio of developed bone metastasis was less than 20% in the premenopausal mice model, while the ratio increased to over 80% in mice with OVX-induced bone loss [44]. Administration of OPG-Fc, a potent inhibitor of osteoclastogenesis that prevents RANKL-RANK binding, reversed OVX-induced bone loss and thus suppressed the reproliferation of dormant breast tumour cells *in vivo* [48]. Additionally, oestrogen depletion triggered oestrogen receptor (ER)-positive breast tumour cell awakening from dormancy by activating angiopoietin-2 (ANGPT2) signalling in the bone marrow niche [45]. In men, androgen deprivation therapy is well known to cause bone loss. Another study conducted by Ottwell and colleagues mimicked the effects of androgen deprivation by castrating 12-week-old BALB/c nude mice, which caused increased bone resorption and loss of bone volume. The results showed that castration triggered a significantly higher rate of bone metastasis of disseminated PC-3 cells compared to the sham operation group [46]. In fact, androgen deprivation and OVX are critical therapies for managing aggressive and advanced prostate cancer and ovarian cancer, respectively. Paradoxically, the bone loss caused by androgen or oestrogen insufficiency increased the risks of overt tumour metastasis or relapse in the bone [49–51]. The influence of this undesirable adverse effect should be taken into consideration in future clinical investigations.

Bone metastasis tumour cells exit from dormancy can be influenced by factors produced during osteoclast-mediated bone resorption, including TGFβ1 and MMP-9. To reactivate themselves from dormancy, dormant tumour cells are inclined to promote osteoclastogenesis by upregulating the expression of several receptors, such as VCAM-1 and PTHrP. Hormone-related therapies (OVX, oestrogen withdrawal, and androgen deprivation) could facilitate osteoclastogenesis and increase bone resorption, subsequently releasing dormant tumour cells to reproliferate and consequently inducing overt metastasis. Therapeutic agents that suppress bone resorption could inhibit bone metastasis, such as EGFR tyrosine kinase inhibitor, Cathepsin K inhibitor, Cathepsin B inhibitor. TGF, transforming growth factor; MMP-9, matrix metalloproteinase-9; VCAM-1, vascular cell-adhesion molecule 1; PTHrP, parathyroid hormone-related protein; IL-1β, interleukin-1β; IL-1R, interleukin-1 receptor; PGE2, prostaglandin E2; RANK, receptor activator of nuclear factor-κB; RANKL, receptor activator of nuclear factor-κB ligand; sRANKL, a soluble form of the RANKL; TNF-α, tumour necrosis factor-α; OPG, osteopontin; EGFR, epidermal growth factor receptor. Graphics were partly generated using Servier Medical Art, provided by Servier, licensed under a Creative Commons Attribution 3.0 unported license (https://smart.servier.com/).

### Self-control of dormant tumour cells by utilizing bone remodelling

It is well documented that bone remodelling could largely impact tumour cell dormancy by either maintaining them in a dormant state or reactivating them from dormancy. However, limited attention has been focused on how dormant tumour cells maintain their dormant state or even reactivate themselves during bone remodelling [52].

For the sake of reactivating themselves from dormancy, dormant tumour cells are inclined to promote osteoclastogenesis to facilitate bone resorption (Fig. 2) (Table 3). For example, breast tumour cells overexpress vascular cell-adhesion molecule 1 (VCAM-1), a member of the transmembrane immunoglobulin superfamily. The excess VCAM-1 increased the recruitment of osteoclast precursors via directly interacting with the cognate receptor integrin α4β1, thus stimulating their adhesion to dormant breast tumour cells. Eventually, activated osteoclastogenesis enhanced bone resorption and reawakened dormant breast tumours to regrowth. Treatment with antibodies against VCAM-1 and integrin α4 effectively suppressed bone metastasis progression and preserved bone structure [24]. Tumour-derived parathyroid hormone-related protein (PTHrP) can stimulate osteoclastogenesis and subsequent bone resorption by stimulating RANKL expression in an autocrine manner [53]. Moreover, PTHrP-overexpressing tumour cells could block pro-dormancy gene expression, suggesting that PTHrP also plays a role in promoting tumour cell exit from dormancy [29]. These findings indicate that identifying tumour-derived osteoclastogenic factors, such as VCAM-1 and PTHrP, might provide new potential therapeutic targets for preventing and inhibiting metastatic recurrence in bone.

**Table 3:** Self-control of dormant tumour cells by utilizing bone remodelling

Self-control	Dormant cancer types	Reactions	Mechanisms	Reference
Reactivate from dormancy	Breast cancer cells	↑ VCAM-1	↑ the recruitment of osteoclast precursors; directly interacting with the cognate receptor integrin α4β1; ↑ preosteoclasts’ adhesion to dormant breast tumour cells	[24]
	Breast cancer cells and giant cell tumour of bone	↑ PTHrP	↓ pro-dormancy gene expression; ↑ osteoclastogenesis and bone resorption through ↑ RANKL expression in an autocrine manner	[29, 53]
Maintain dormancy	Breast cancer cells	↑ N-Cadherin	↑ the adhesive attraction of MDA to SNOs	[54]
	Prostate cancer cells	↑ ROR2	↑ Wnt5a-induced dormancy driven by osteoblasts	[28]
	Prostate cancer cells	↑ Axl	Gas6/Axl signalling ↑ expression of TGFβ2 and its receptor	[55, 56]
	Prostate cancer cells	↑ TBK1	After binding to osteoblasts,↓ mTOR and ↑ drug resistance to chemotherapy	[57]
	Acute lymphoblastic leukemia cells	Express OPN receptors	Compete with HSCs for adhesion to OPN within the bone marrow	[23]
	Acute lymphoblastic leukemia cells	Express CXCR4	Compete with HSCs for binding to CXCL12 in the bone marrow	[58, 59]
	Prostate cancer cells and myeloma cells	Express Annexin II receptor	Compete with HSCs for binding to annexin II on osteoblasts	[32, 60, 61]
	Prostate cancer cells	N/A	↑ haematopoietic differentiation, replace and mobilize HSCs from the osteoblastic niche	[6]

*Note*: ‘↑’ represents increased, upregulated, induced, accelerated, enhanced and activated; While ‘↓’ represents decreased, downregulated, inhibited; *N/A* represents not applicable

On the other hand, dormant tumour cells preferentially adhere to osteoblasts, thus facilitating bone formation to induce and maintain themselves in a dormant state (Fig. 1) (Table 3). A very recent study found that high expression of N-cadherin in MDA-MB-231 (MDA) breast cancer cells reduced tumour metastasis and bone osteolysis in a mouse model. Mechanistically, N-cadherin facilitated the adhesive attraction of MDA to SNOs *in vitro*, allowing SNOs to induce dormancy in MDA cells [54]. These evidences may partially explain why dormant tumour cells were often located near the endosteal niche with enriched SNOs. Alternatively, dormant tumour cells could also aberrantly increase the expression of several genes, such as ROR2, Axl and TANK binding kinase 1 (TBK1), to facilitate osteoblast-induced tumour cell dormancy. Ren et al. showed that silencing ROR2 in dormant prostate tumour cells abrogated Wnt5a-induced dormancy driven by osteoblasts during bone metastasis [28]. Dormant DTCs residing close to osteoblasts have been proven to express high levels of Axl, which inspired growth suppression of prostate cancer cells by osteoblasts [55]. The upregulated expression of Axl could combine with the growth-arrest specific 6 (Gas6) produced by osteoblastic cells, which induced the expression of TGFβ2 and its receptor in the endosteal niche [56]. Similarly, prostate tumour cells increased their expression of TBK1 to interact with and inhibit mammalian/mechanistic target of rapamycin (mTOR) when binding to osteoblasts. Eventually, the inhibition of mTOR signalling induced prostate tumour dormancy and contributed to chemoresistance *in vitro* and *in vivo* [57].

One hypothesis that has gained favor is that the process of DTCs colonizing osteoblastic niche is functionally similar to the homing behavior of HSCs. The presence of HSCs hindered DTC colonization of bone niche in a competitive manner and thus limited bone metastasis [6]. There are three ways of DTCs compete with HSCs in occupying the osteoblastic niche, thus initiating and maintaining themselves in a dormant state [26]. Firstly, osteoblasts secrete molecules that are critical in HSCs homing, such as OPN and C-X-C motif chemokine ligand 12 (CXCL12), which are also utilized by DTCs to establish footholds in the bone marrow [23, 58, 59]. Secondly, DTCs mimic HSC-like phenotype, allowing them competitively bind to the proteins, such as annexin II, that are responsible for HSCs’ bone localization [32, 60, 61]. Finally, DTCs could directly or indirectly drive HSCs’ maturity, and displace HSCs from the osteoblastic niche. Disseminated prostate cancer cells accelerated haematopoietic differentiation, thus replacing and mobilizing HSCs from the osteoblastic niche into the peripheral blood [6]. Although dormant tumour cells may not be directly intervened by HSCs, the presence of HSCs largely restricted the occupancy of DTCs to the osteoblastic-induced dormancy microenvironment. Therefore, targeting HSCs is a promising therapeutic strategy for limiting bone metastasis.

Collectively, the ability of dormant tumour cells utilizing bone remodelling or competing with HSCs to control their dormant state is non-negligible, as they might further complicate the consequences after primary therapies. However, how dormant cells determine whether they should become dormant or reawakened remains largely unknown and deserves more profound mechanistic studies in the future.

### The role of inflammatory cytokines in regulating tumour cell dormancy during bone remodelling

The “bone-tumour-inflammation network” is a system that tightly combines the bone microenvironment with the tumour microenvironment through inflammatory responses. However, the fact that inflammatory cytokines produced during bone remodelling participate in regulating tumour cell dormancy is seriously underestimated. In this section, we illustrated some vital inflammatory cytokines produced during bone remodelling (mainly by osteoblasts) that could determine the conditions of tumour dormancy.

Leukaemia inhibitory factor (LIF), an inflammatory cytokine of the IL-6 family, is produced by osteoblasts. LIF promoted breast cancer cell dormancy in the bone by binding to the LIF receptor (LIFR), which induced the expression of crucial dormancy-related genes in breast tumour cells, including thrombospondin-1 (TSP1), tropomyosin-1 (TPM1), TGFβ2, prolyl 4 hydroxylase α-1 (P4HA1), miRNA-190 (miR-190) and SELENBP1. Knockdown of LIFR resulted in lower p53 protein levels and greater c-MYC and pSRC (Y527) protein levels, suggesting that LIFR was crucial for MCF7 cells to remain dormant [29]. CXCL12, another chemokine secreted by osteoblasts, triggers DTC dormancy in the bone marrow by binding to C-X-C motif chemokine receptor 4 (CXCR4), one of the receptors of CXCL12 in tumour cells [31]. Moreover, a high concentration of CXCL12-induced tumour dormancy contributes to drug resistance, arousing the clinical value of controlling CXCL12 production and scavenging excessive CXCL12 in the bone marrow [30]. Evidence has proven that Gas6 produced by osteoblasts can also induce tumour dormancy during bone metastasis [32]. Decker et al. revealed that the sympathetic nervous system/norepinephrine (NE) reactivated dormant prostate tumour cells in the bone marrow niche by downregulating the expression of Gas6 in osteoblasts [62]. Gas6 also inhibits the production of tumour necrosis factor (TNF)-α, interleukin (IL)-1β and IL-6 in monocytes and macrophages, which are essential pro-inflammatory factors [63]. Therefore, Gas6 plays a dominant role in connecting tumour dormancy and bone microenvironment with the inflammation network.

In contrast to the dormancy-inducing effect of LIF, CXCL12 and Gas6, the inflammatory cytokine IL-1β promoted tumour proliferation and subsequently triggered overt metastasis of breast tumour cells. Direct interaction between breast tumour cells and osteoblasts promoted IL-1β release from both cell types, which enhanced the progression of EMT, invasion, migration, angiogenesis, and bone colonization [64]. Moreover, inhibiting its receptor (IL-1R) signalling by anakinra, an IL-1R antagonist, impeded overt metastasis by maintaining disseminated breast tumour cells in dormancy [65]. Similarly, Sosnoski and colleagues found that IL-1β and TNF-α broke the dormant state of breast tumour cells induced by osteoblasts in their coculture system. These two cytokines stimulate the production of prostaglandin E2 (PGE2), another critical inflammatory molecule, leading to the nuclear localization of Ki67 in breast tumour cells [25].

Overall, inflammatory cytokines produced mainly by osteoblasts during bone remodelling play a dual role in tumour cell dormancy. This may partially explain why osteoblasts could both induce tumour cell dormancy and promote tumour cell growth in the bone marrow. Inflammatory factors produced during the interactions between dormant tumour cells and osteoblasts may serve as novel biomarkers, which can be utilized to predict the state of tumour cell dormancy and the potential risk of tumour relapse.

### Clinical relevance

There is compelling clinical evidence that tumour cell dormancy exists and that the bone microenvironment may initiate tumour cell dormancy and trigger DTC proliferation. Increasing the understanding that osteoblasts and osteoclasts control the state of dormancy provides essential clinical implications. For instance, identifying dormancy-related factors produced by osteoblasts and osteoclasts provides opportunities to predict and prevent dormant tumours from developing overt bone metastasis. Bone-disseminated prostate tumour cells with lower expression of TGFβRIII caused by differentiated osteoblasts might be less able to enter dormancy. Further prognostic analysis confirmed that lower levels of TGFβRIII expression are associated with metastasis and poor clinical performance outcomes in prostate cancer patients [20]. ROR2 could be used as a reliable biomarker in predicting bone metastasis in prostate cancer patients. The bone marrow of patients with low levels of ROR2 disrupted Wnt5a signalling produced by the osteoblastic niche, which hinted at the potential development of osteolytic tumours in bone [28]. Additionally, a significant and positive correlation between high N-cadherin levels produced by osteoblasts and overall survival was identified in oestrogen- or progesteron-positive breast tumour patients [54]. Increased bone resorption is also related to tumour burden in patients. Lawson et al. proved that the serum level of the bone resorption biomarker C-terminal telopeptide (CTX) was strongly correlated with β2-microglobulin (β2m), a protein that can measure tumour burden in newly diagnosed myeloma patients (*R*^2^=0.3588, *P*<0.0001) [21].

Another important implication may come in the context of treatment. Because dormant tumour cell-targeting agents are not yet clinically available, treatments that regulate bone biological processes, especially anti-resorptive therapies, hold promise for controlling tumour dormancy (Fig. 2). In the clinic, bisphosphonate treatments, such as zoledronic acid, decreased bone resorption and limited inadvertent activation of dormant breast cancer cells in the bone of patients [66–68]. Recent clinical trials exhibited similar effects on preventing tumour bone metastasis by treatment with the anti-RANKL agent denosumab, which disrupts the RANK-RANKL signalling involved in osteoclastogenesis [69–72]. The epidermal growth factor receptor (EGFR) signalling was found to regulate the production of pro-osteoclastogenic factors and osteoclast formation [73]. Gefitinib, a clinically available EGFR tyrosine kinase inhibitor, reduces osteoclast differentiation and blocks osteolytic bone metastasis [74, 75]. Cysteine cathepsins are a class of proteolytic enzymes that function in numerous physiological processes and have emerged as drug targets in bone-related diseases [76]. Cathepsin K (CK), one of the members, is predominantly secreted by mature osteoclasts to facilitate bone degradation [77, 78]. Odanacatib, a CK-selective inhibitor, has been evaluated in a phase II trial for breast-to-bone metastasis treatment and showed some positive results accompanied with decreased bone resorption before discontinuation of clinical testing [76]. Another cysteine family member, Cathepsin B (CB) is closely associated with local recurrence and distant metastases in patients with bone chondrosarcoma [79]. Although CB plays less important role in regulating osteoclastogenesis than CK, inhibition of CB activity is also shown to impede bone metastasis progression [80, 81]. Withana et al. proved that selective CB inhibitor, CA-074, significantly decreased late-stage bone metastasis of breast cancer [82]. Besides, inhibitors that suppress bone resorption, such as dasatinib (Src tyrosine kinase inhibitor) and PSK-1404 (integrin αVβ3 inhibitor) were proved to inhibit osteolytic bone metastasis [83–86]. Yet, the precise mechanisms or long-term outcomes of these inhibitors in cancer patients warrant further evaluation. Taken together, these evidences suggest that suppression of bone resorption may prevent the reactivation of dormant tumour cells in the bone marrow niche before the development of overt metastatic tumours.

## Conclusion and future prospects

Undoubtedly, cellular dormancy is a highly complex phenomenon involved during bone metastasis. The interactions between osteoclast/osteoblast-mediated bone remodelling and dormant tumour cells are likely among the most critical rationales of metastatic tumour outgrowth in bone. A majority of strategies focus on keeping dormant tumours ‘asleep’ instead of ‘waking them up’ so that tumour outgrowth and metastasis can be potentially prevented. However, we cannot exclude the possibility that creating more bone niches for supporting dormant tumour cell survival will cause long-term deleterious effects. Dormant tumour cells are resistant to most chemotherapies and radiotherapies that target proliferative tumour cells [16]. Activating dormant tumour cells to succumb to conventional cancer treatment could serve as an alternative anticancer strategy. Lawson et al. suggested that activating dormant myeloma cells might render them more susceptible to the existing anticancer agents, thereby overcoming drug resistance and achieving complete remission [21]. However, this treatment approach has been widely debated because preclinical evidence has shown that activated dormant tumour cells exhibit enhanced proliferation and metastasis capacities [10]. It is currently difficult to determine the pros and cons between these two strategies during bone metastasis. More systematic and in-depth mechanistic studies are urgently required before this knowledge can be used for therapeutic benefit [87]. Since inhibitor of apoptosis proteins (IAPs) are overexpressed in many human malignancies, IAPs antagonists have emerged as potent anticancer drug candidates [88]. The major anticancer mechanisms of IAPs antagonists involve disrupting IAPs interaction with caspases and decreasing intracellular levels of IAPs [89]. However, several evidence showed that IAPs antagonist treatment unexpectedly increased bone metastasis. Owing to the osteoclastogenesis that was promoted by IAPs antagonists through non-canonical NF-κB pathway, dormant tumour cells in the bone marrow were reactivated [90, 91]. Therefore, co-treatment with anti-resorptive agents, such as zoledronic acid, could potentially prevent such unwanted side effects of IAPs antagonists on bone metastasis [92]. Moreover, the generally immune-privileged nature of the bone can be favourable for dormant tumour cell seeding and escape from immune surveillance. This phenomenon potentially explains why bone is such a common site for both dormant and active tumour cells to anchor. As immunotherapies continue to gain momentum for tumour treatment in the clinic, an exquisite understanding of tumour-bone-immune crosstalk, especially on how current therapies affect bone metastatic tumour cells, will shed light on new anticancer strategies.

## Data Availability

Not applicable.

## Abbreviations

CTCs: Circulating tumour cells
DTCs: Disseminated tumour cells
EMT: Epithelial-to-mesenchymal transition
TGF: Transforming growth factor
SNOs: Spindle-shaped N-cadherin + /CD45- osteoblasts
OPN: Osteopontin
NSG: NOD-SCID IL2Rγ(null)
GFP: Green fluorescent protein
BMP7: Bone morphogenetic protein 7
BMP1: Bone morphogenetic protein 1
MAPK: Mitogen-activated protein kinases
RB: Retinoblastoma
PTH: Parathyroid hormone
ECM: Extracellular matrix
MMP-9: Matrix metalloproteinase-9
RANK: Receptor activator of nuclear factor-κB
RANKL: Receptor activator of nuclear factor-κB ligand
sRANKL: A soluble form of the RANKL
ER: Oestrogen receptor
OVX: Ovariectomy
ANGPT2: Angiopoietin-2
VCAM-1: Vascular cell-adhesion molecule 1
PTHrP: Parathyroid hormone-related protein
TBK1: TANK binding kinase 1
Gas6: Growth-arrest specific 6
LIF: Leukaemia inhibitory factor
LIFR: LIF receptor
TSP1: Thrombospondin-1
TPM1: Tropomyosin-1
P4HA1: Prolyl 4 hydroxylase α-1
miR-190: MiRNA-190
CXCL12: C-X-C motif chemokine ligand 12
CXCR4: C-X-C motif chemokine receptor 4
NE: Nervous system/norepinephrine
TNF-α: Tumour necrosis factor-α
IL-1β: Interleukin-1β
IL-1R: Interleukin-1 receptor
PGE2: Prostaglandin E2
CTX: C-terminal telopeptide
β2m: β2-microglobulin
mTOR: Mammalian/mechanistic target of rapamycin
DKK3: Dickkopf-related protein 3
ROR2: Receptor tyrosine kinase-like orphan receptor 2
NDRG1: N-myc downstream-regulated gene 1
EGFR: Epidermal growth factor receptor
CK: Cathepsin K
CB: Cathepsin B
